# Exploring the potency of polyphenol-rich blend from *Lonicera caerulea* var. *Kamtschatica* sevast., *Aronia melanocarpa*, and *Echinacea purpurea*: Promising anti-inflammatory, antioxidant, and antiviral properties

**DOI:** 10.1016/j.heliyon.2024.e35630

**Published:** 2024-08-02

**Authors:** Katarzyna Zima, Barbara Khaidakov, Marta Sochocka, Michał Ochnik, Krzysztof Lemke, Paulina Kowalczyk

**Affiliations:** a AronPharma Ltd. R&D Department, Trzy Lipy Street 3, 80-172, Gdańsk, Poland; b Department of Physiology, Medical University of Gdańsk, Dębinki 1 Street, 80-211, Gdańsk, Poland; c Laboratory of Virology, Hirszfeld Institute of Immunology and Experimental Therapy, Polish Academy of Sciences, Weigla 12 Street, 53-114, Wrocław, Poland; d 3P-Medicine Laboratory, Medical University of Gdańsk, Dębinki 7 Street, 80-211, Gdańsk, Poland

**Keywords:** Polyphenol, Immunomodulation, Antioxidant, Plant extracts, Antiviral activity

## Abstract

Previous studies have highlighted the beneficial properties of plants rich in polyphenols, such as *Lonicera caerulea* var. *Kamtschatica* Sevast. (LCK), *Aronia melanocarpa* (AM), and *Echinacea purpurea* (EP). These plants have demonstrated antioxidant, immunomodulatory, and potential antiviral effects. Thus, the objective of this study was to investigate the impact of the ELA blend, a polyphenol-rich blend containing EP, LCK, and AM, on the cellular mechanisms involved in viral infection. To assess the effects of the ELA blend, various experiments were conducted using A549 cells and a mucociliary tissue 3D model called EpiAirway™. Inflammation and oxidative stress induced by LPS were evaluated through measurements of SOD activity, ELISA, and qPCR analysis. Additionally, antiviral assays were performed in a cell-present environment to examine the blend's effectiveness against HCoV-OC43. The results showed that the ELA blend-treated group exhibited reduced expression of *IL1B*, *CXCL8*, *ICAM1*, *MCP1*, and *RELA* in both A549 cells and EpiAirway™. Moreover, the blend enhanced the expression of *CAT*, *HMOX1*, *SOD1*, and *SOD2* in A549 cells. The antiviral activity of the ELA blend was also investigated, i.e. its influence on viral replication cycle, to determine the potential as an antiviral preparation. At the highest non-cytotoxic concentration, the ELA blend demonstrated a 87.5 % reduction in viral titer when administered simultaneously with HCoV-OC43. It emphasize potential ability of the preparation to block viral entry to the host cells. At the same time, ELA blend did not express virucidal activity, i.e. inactivation of free viral particles, against HCoV-OC43. In conclusion, ELA blend displayed antiviral activity and exhibited immunomodulatory and antioxidant effects. Based on these findings, it can be concluded that ELA blend has potential for the prevention and treatment of viral infections.

## 1 Introduction

Infectious disease outbreaks have been listed as one of the top 13 worldwide critical health concerns for the next decade by the World Health Organization (WHO) [1]. Upper respiratory tract infections (URTI) impose an enormous economic burden due to health system overload and missed educational or professional activities. URTI are most often caused by viruses, such as: rhinovirus, coronavirus, influenza virus, adenovirus, and enterovirus [2]. Drugs commonly used to treat URTI are solely meant to provide symptomatic relief. So far, we have a restricted arsenal of antiviral agents. Moreover, these substances have limited effectiveness and significant side effects [3]. Due to the outbreak of the COVID-19 pandemic, there is an increased demand for new treatments, including natural substances with high bioavailability, immunoregulatory and antiviral properties. Plant-derived, rich in active agents, products are increasingly being recognized as an important additional treatment and alternative to standard medications [4]. However, health benefits are strongly related to both active agents content (e.g., polyphenols) and bioavailability, which are dependent on their origin, processing, and growing conditions. The evidence from a number of studies has shown antioxidant, immunomodulatory, and potentially antiviral effects of *Lonicera caerulea* var. *Kamtschatica* Sevast. (called haskap berries; LCK), *Aronia melanocarpa* (called black chokeberry; AM), and *Echinacea purpurea* (L.) Moench (called purple coneflower; EP).

LCK has been found to have antioxidant and anti-inflammatory effects [5,6]. Though direct evidence on the effect of LCK on URTI is scarce, studies on related varieties like *Lonicera japonica* suggest anti-URTI properties [7]. Additionally, *Lonicera caerulea* var. *Emphyllocalyx* extract has shown immune modulation in murine models of *Streptococcus pyogenes* infection [8]. Hence, while direct evidence is lacking, it is plausible that LCK may exert similar effects to other *Lonicera caerulea* varieties. AM, or black chokeberry, exhibits promising effects on URTI. Recent studies demonstrate its antiviral activity against SARS-CoV-2 and influenza viruses. Rich in phenolic compounds like anthocyanins and chlorogenic acids, AM shows potent antioxidative properties, potentially reducing oxidative stress and inflammation in the respiratory system [9]. Research suggests that AM inhibits pro-inflammatory cytokines and reactive oxygen species production in lung epithelial cells, while also displaying effectiveness against various influenza strains, including oseltamivir-resistant ones. Products like Bioaron®C, containing AM extract, exhibit broad-spectrum antiviral activity against URTI-causing viruses, indicating AM's therapeutic potential [10]. The efficacy of EP roots and herbs to alleviate symptoms of upper respiratory illnesses is widely documented. Numerous studies have demonstrated that EP enhances innate immunity by stimulating neutrophils, macrophages, granulocytes, and natural killer cells [[11], [12], [13], [14], [15], [16], [17], [18]]. EP has an antiviral effect on virally confirmed colds and shows maximal effects on recurrent infections and is used as infection prevention [19]. Administration of EP extract in acute URTI may be beneficial in the early stages of the disease [20]. However, a recent meta-analysis found no evidence that administration of EP affects the duration of URTI [21].

While prior studies have illuminated the beneficial attributes of these plants, the focus of this investigation lies in elucidating the impact of the ELA blend—a polyphenol-rich blend comprising EP, LCK, and AM—on the cellular mechanisms pertinent to viral infections of the upper respiratory tract. In this study, we aimed to assess the specific effects of the ELA blend on LPS-induced inflammation and oxidative stress using both 2D (A549 cells) and 3D (EpiAirway™) models. Additionally, we examined its efficacy in inhibiting the binding of SARS-CoV-2 spike receptor-binding domain (RBD) to angiotensin-converting enzyme 2 (ACE2) receptors *in vitro*. Furthermore, our investigation extended to evaluating the antiviral potential of the ELA blend against human respiratory betacoronavirus-1 (HCoV-OC43) within a cell-present setting.

Our findings demonstrate that the ELA blend exhibits immunomodulatory, antioxidant, and direct antiviral effects. Specifically, we observed reduced expression of inflammatory markers and enhanced antioxidant enzyme activity in ELA blend-treated cells. Moreover, the blend significantly inhibited viral replication of HCoV-OC43. These results suggest the potential of the ELA blend as a preventive and therapeutic agent against viral infections.

## 2 Materials and methods

### 2.1 Blend preparation and analysis

The EP, LCK and AM extracts were provided by GREENVIT Ltd (Zambrów, Poland). Pure extracts were produced by water or ethanol extraction in mild conditions in order to preserve thermolabile compounds. The liquid extracts were concentrated in vacuum and spray dried. Pure extracts were standardized as follows: LCK with a minimum of 15 % anthocyanins, AM with a minimum of 25 % anthocyanins, EP root and EP herb with a minimum of 2 % chicoric acid. The ELA blend has been standardized to contain a minimum of 15 % anthocyanin content as measured by HPLC, a minimum of 30 % polyphenol content as measured by UV, and a minimum of 0.2 % chicoric acid content as measured by HPLC. The components were blended in precise ratios to fulfill the stated criteria for standardizing the final product. The mixture is a confidential formula developed through a project carried out by AronPharma Ltd. (Gdańsk, Poland). Designed through the methodology of Design of Experiments (Design-Expert®, Stat-Ease®, Minneapolis, MN, USA), the blend is currently pending patent approval.

The content of anthocyanins was determined by the ACQUITY UPLC I-Class PLUS System liquid chromatograph with a PDA detector (Miliford, MA, USA). Standard of cyanidin 3-galactoside was purchased from PhytoLab GmbH & Co. KG (Vestenbergsgreuth, Germany). Reference standard of cyanidin-3-O-glucoside chloride was supplied by USP Reference Standards (Basel, Switzerland). Standard of cyanidin 3-O-arabinoside chloride was purchased from Extrasynthese (Lyon, France). The remaining anthocyanins were identified by the use of published research articles [[22], [23], [24]]. The chromatograms were recorded at 520 nm. Before analysis, the samples of the blend were dissolved in a mixture of 0.01 % formic acid in methanol:water (8:2, v/v). Chromatographic separation of the analytes was achieved on an Acquity UPLC® BEH C18 column (2.1 mm × 150 mm × 1.7 μm) without any pre-column. The column was thermostatted at 35 °C. The temperature of the autosampler was set to 5 °C. Mobile phase consisted of 2 % formic acid in water (v/v, component A) and acetonitrile (component B). The flow rate of the mobile phase was set to 0.3 mL/min. The mobile phase eluted under the following conditions (shown in relation to the component A): initial to 3.0 min isocratic: 96 %, next gradient: 3.0 min–96 %, 8.0 min–85 %, 11.0 min–20 %, 11.5 min–96 %. The equilibration time was 1.5 min. Data acquisition and result analysis were performed using the Empower Chromatography Data System by Waters.

The total polyphenols content (TPC) was determined by the colorimetric Folin-Ciocalteu method with caffeic acid (Sigma-Aldrich, Saint Louis, MO, USA) as a standard using a 6-point calibration curve. Two blend samples were diluted in methanol: water (4:6 v/v) to reach a concentration of 1 mg/mL prior to the measurements. 1 mL of the blend was combined with 4 mL of water and 0.5 mL of Folin–Ciocalteu reagent (Sigma-Aldrich, Saint Louis, MO, USA). After 1 min, 2 mL of 20 % (w/v) sodium carbonate aqueous solution was added to a 10 mL volumetric flask and filled with water to a mark. The samples were incubated at room temperature for 30 min in the dark. NanoPhotometer® NP80 spectrophotometer (Implen GmbH, München, Germany) was used to measure the absorbance spectrophotometrically at 760 nm.

The differential pH method based on spectrophotometric technique was used to measure the anthocyanin content in the blend sample. Two blend samples were initially diluted in a methanol mixture (4:6 v/v) to achieve a concentration of 0.1 mg/mL. Subsequently, samples were diluted with buffer solutions to an appropriate concentration that falls within the linear range of the spectrophotometer - 1 mL of each blend solution was diluted with pH 1.0 buffer (0.025 M potassium chloride) and pH 4.5 buffer (0.4 M sodium acetate) in 10-mL volumetric flasks. Before using, the pH of buffer solutions was measured using the pH meter and adjusted to accurate pH using HCl. Then, the flasks were left to sit at room temperature in the dark for 5 min. Absorbance measurements were taken within 15 min of samples preparation. NanoPhotometer® NP80 spectrophotometer (Implen GmbH, München, Germany) was used to measure the absorbance spectrophotometrically at 520 nm and 700 nm. Using the molecular weight of cyanidin-3-glucoside and the molar absorbance coefficient, the anthocyanin concentration was determined.

### 2.2 Cell lines

The human lung epithelial carcinoma cell line (A549; ECACC 86012804) was purchased from the European Collection of Authenticated Cell Cultures (ECACC, Porton Down, England, UK). A549 cells were cultured in Nutrient Mixture Kaighn's Modification Medium (F12K; Gibco, Waltham, MA, USA) containing 10 % Fetal Bovine Serum (FBS; Corning, Corning, NY, USA) and 1 % antibiotic-antimycotic solution (A/A; Sigma-Aldrich, Saint Louis, MO, USA). Human ileocecal adenocarcinoma cells (HCT‐8 (HRT‐18); ATCC® CCL‐244™) were purchased from the American Type Culture Collection (ATCC, Manassas, VA, USA). HCT-8 cells were cultured in Roswell Park Memorial Institute medium (RPMI 1640; HIET PAS, Wroclaw, Poland) supplemented with 10 % FBS (Sigma‐Aldrich, Steinheim, Germany). The cells were incubated at 37 °C in a humidified atmosphere of 5 % CO_2_.

### 2.3 *In vitro* lung tissue model

The EpiAirway™ three-dimensional human airway epithelium tissue model (AIR-100) was purchased from MatTek, Corp. (Ashland, MA, USA). The model consisted of fully differentiated human airway epithelium grown at the air-liquid interface. Upon receipt, the tissues were transferred to 6-well culture plates containing 1 mL of fresh cell culture medium (AIR-100-MM; MatTek, Ashland, MA, USA) and were placed in the tissue culture incubator set at 37 °C, 5 % CO_2_ for 18–24 h prior to experiments.

### 2.4 Experimental design

The stock solution of the ELA blend at a concentration of 50 mg/mL was prepared in 40 % DMSO, and filtered through a Millipore 0.22 μm filter, and then brought to a working concentration in the culture medium. The working solution was prepared each time before the experiment.

Confluent A549 cells at passage numbers 7–11 or EpiAirway™ tissues were divided into the following groups: control group, LPS-treated group, and ELA blend + LPS-treated group. The control group was incubated with only the culture medium without any additional interventions or treatments.

To evaluate the impact of LPS on the activity of A549 cells, 1 μg/mL of LPS (Sigma-Aldrich, Saint Louis, MO, USA) was added to the cells for a duration of either 2 or 24 h. In order to investigate the effect of the ELA blend on LPS-induced inflammation in A549 cells, the cells were first pretreated with 250 μg/mL of ELA blend for either 2 h or 4 h. After the pretreatment, the cells were then incubated with 1 μg/mL of LPS for an additional 2 h or 24 h, respectively. To evaluate the impact of LPS on the activity of EpiAirway™ tissues, 1 μg/mL of LPS was added to the basal layer of the tissue cultures for a duration of 24 h. To investigate the effect of the blend on LPS-induced inflammation in EpiAirway™ tissues, a pretreatment was performed using 50 μg/mL of ELA blend for 4 h (added to the basal layer of the tissues), followed by incubation with 1 μg/mL of LPS for 24 h.

Thereafter, the cultures were designated for further analysis, including the measurement of Interleukin (IL)-8, IL-6, and Superoxide Dismutase (SOD) levels, as well as the expression of selected genes.

### 2.5 Cytokine production

For each experimental group, cell culture medium of A549 cells and EpiAirway™ tissues was collected for further analysis. IL-8 and IL-6 protein levels were measured using specific ELISA – DuoSet® ELISA Development Systems (R&D Systems, Inc., Minneapolis, MN, USA) according to the manufacturer's instructions. Absorbance was read at 450 nm and 570 nm (for background) on the PerkinElmer EnVison 2103 Multilabel Reader (PerkinElmer, Waltham, MA, USA).

### 2.6 SOD activity

For each experimental group, cell culture medium of A549 cells and EpiAirway™ tissues was collected for further analysis. SOD activity was measured using the SOD Activity Assay Kit (Sigma-Aldrich, Saint Louis, MO, USA) according to the manufacturer's instructions. Absorbance was read at 450 nm using the PerkinElmer EnVison 2103 Multilabel Reader (PerkinElmer, Waltham, MA, USA). The standard curve was developed to convert the raw absorbance values into concentrations expressed in [units/mL], ensuring accurate and reliable quantification.

### 2.7 Gene expression

Total RNA was prepared from A549 cells and EpiAirway™ tissues with the Total RNA Mini kit (A&A Biotechnology, Gdańsk, Poland). The quantity and purity of the RNA samples were determined by measuring the absorbance ratios at 260/280 nm and 260/230 nm with a NanoPhotometer® NP80 spectrophotometer (Implen GmbH, München, Germany). The RNA concentration was calculated based on the absorbance at 260 nm (A260), using the formula: RNA concentration (μg/mL) = A260 × dilution factor × 40. Purity was assessed by calculating the 260/280 nm and 260/230 nm absorbance ratios, with acceptable values being ∼2.0 and 2.0–2.2, respectively. 1 μg RNA was transcribed into cDNA using the High-Capacity cDNA Reverse Transcription Kit (Applied Biosystems™, Waltham, MA, USA). qRT-PCR was performed using the PerfeCTa SYBR® Green FastMix Reagent (Quantabio©, Beverly, MA, USA) and the Rotor-Gene Q real-time cycler (Qiagen, Hilden, Germany). The total PCR volume used in our study was 10 μL. The PCR cocktail comprised the following components: 5 μL of SYBR PerfeCTa SYBR® Green FastMix Reagent (Quantabio©, Beverly, MA, USA); 0.25 μL of forward primer (10 μM); 0.25 μL of reverse primer (10 μM); 0.5 μL of cDNA template; 4 μL of nuclease-free water kit (A&A Biotechnology, Gdańsk, Poland). The primers used in our study (Table 1) were designed by the authors using Primer-BLAST (National Center for Biotechnology Information, Bethesda, MD, USA) and subsequently synthesized by Genomed Ltd. (Warsaw, Poland). The PCR protocol included an initial denaturation step at 95 °C for 30 s; followed by 40 cycles of 95 °C for 5 s, 60 °C for 20 s. The melting curve and agarose gel analysis were used to ensure the specificity of the PCR products and to detect any potential primer-dimer formations or non-specific amplifications. *GAPDH* (glyceraldehyde-3-phosphate dehydrogenase) and *TBP* (TATA-binding protein) were used as refence genes, and the relative quantification was determined using the 2^−ΔΔCt^ method. The 2^-ΔΔCt^ method, also known as the comparative CT method, compares the CT (cycle threshold) values of the target gene to those of reference genes, normalizing to a control sample to calculate relative gene expression levels [25].

**Table 1:** The primers sequences used in the study.

Gene symbol	Primer Forward	Primer Reverse
*CAT*	ACAGCAAACCGCACGCTATG	CAGTGGTCAGGACATCAGCTTTC
*CXCL8*	CCAGGAAGAAACCACCGGA	GAAATCAGGAAGGCTGCCAAG
*GAPDH*	TGCACCACCAACTGCTTAGC	GCATGGACTGTGGTCATGAG
*HMOX1*	CCAGCAACAAAGTGCAAGATTC	TCACATGGCATAAAGCCCTACAG
*ICAM1*	CCTTCCTCACCGTGTACTGG	AGCGTAGGGTAAGGTTCTTGC
*IL1B*	TCCCCAGCCCTTTTGTTGA	TTAGAACCAAATGTGGCCGTG
*MCP1*	GAGAGGCTGAGACTAACCCAGA	ATCACAGCTTCTTTGGGACACT
*NFE2L2*	CACATCCAGTCAGAAACCAGTGG	GGAATGTCTGCGCCAAAAGCTG
*NOS3*	GAAGGCGACAATCCTGTATGGC	TGTTCGAGGGACACCACGTCAT
*PTGS2*	CGGTGAAACTCTGGCTAGACAG	GCAAACCGTAGATGCTCAGGGA
*RELA*	TGAACCGAAACTCTGGCAGCTG	CATCAGCTTGCGAAAAGGAGCC
*SOD1*	CTCACTCTCAGGAGACCATTGC	CCACAAGCCAAACGACTTCCAG
*SOD2*	CTGGACAAACCTCAGCCCTA	CCTTGCAGTGGATCCTGATT
*TBP*	CACGAACCACGGCACTGATT	TTTTCTTGCTGCCAGTCTGGAC

### 2.8 Assessment of ELA blend's inhibitory effect on the binding of ACE2 and SARS-CCOV-2 RBD *in vitro*

The ability of the blend to inhibit the binding of ACE-2 to SARS-CoV-2 spike protein was assessed using the COVID-19 Spike-ACE2 kit (CoV-SACE2-1, RayBiotech Inc, Peachtree Corners, GA, US) according to the manufacturer's protocol. The blend was tested at five concentrations (ranging from 10 to 2000 μg/mL) and the inhibitory potential for each concentration was evaluated in duplicate. The analyzed blend was mixed with recombinant ACE2 protein, added to an ELISA plate coated with SARS-CoV2 recombinant S protein RBD and incubated overnight at 4 °C. Unbound ACE2 was removed by washing, and binding was assessed by reacting HRP (horseradish peroxidase)-conjugated anti-ACE2 antibody with 3,3′,5,5′-tetramethylbenzidine (TMB). Absorbance at 450 nm was measured with the PerkinElmer EnVison 2103 Multilabel Reader (PerkinElmer, Waltham, MA, USA).

### 2.9 Virus

Human betacoronavirus OC43 (HCoV‐OC43; *Coronaviridae*, ATCC VR‐1558™) was cultured on HCT‐8 cells in a maintenance medium, RPMI 1640 Medium supplemented with antibiotics (100 U/mL penicillin and 100 μg/mL streptomycin) and 2 mM l‐glutamine, all provided by Corning,(Manassas, VA, United States) and 2 % FBS (Sigma‐Aldrich, Steinheim, Germany). Incubation conditions were 34 °C/5 % CO_2_. The viral titer was expressed as a tissue culture infectious dose (TCID_50_) with a 50 % endpoint, where half of the inoculated host cells exhibited a cytopathic effect (CPE). It was calculated using the Spearman–Kärber method [26]. The CPEs of HCoV‐OC43 were visualized using immunoperoxidase staining.

### 2.10 Microscopic viral cytopathic effects (CPE)

After 4–5 days of incubation (the peak of virus production) under an inverted microscope, viral cytopathic effects (CPEs), i.e. changes in cell morphology caused by virus replication, were observed and assessed. CPEs were observed and evaluated with a five-point rank scale: 0—lack of viral replication (lack of CPEs), 1—viral CPEs in 25 % of the cells, 2—viral CPEs in 50 % of the cells, 3—viral CPEs in 75 % of the cells, and 4–100 % of the cells affected with CPEs. The viral titer was expressed with reference to the TCID_50_. The positive controls were antiviral drugs: molnupiravir (50 μM) and interferon (0.01 μg/mL). To convert the scale of cytopathic effects from the rank scale to percentage, the following equation was used: $CPE\left[\%\right]=\left\{\begin{array}{c} 25\times RS-12.5\;when\;RS\geq 0.5\\ 0\;in\;p.p\text{.} \end{array}\right\}$**,** where: RS-average rank value on CPEs scale**,** in p.p. - read: otherwise.

### 2.11 Immunoperoxidase assay

To better visualize CPE of HCoV‐OC43, an indirect immunoperoxidase assay (IPA) was introduced [27]. The cells (HCT-8) were over-layered by the primary mouse anti‐coronavirus monoclonal antibodies (Merck, Darmstadt, Germany) and secondary goat anti‐mouse antibodies conjugated to horseradish peroxidase (Thermo Fisher, Rockford, IL, USA). A virus‐specific presence color reaction was obtained by adding a 3,3′‐Diaminobenzidine (DAB/H_2_O_2_) solution (Sigma, St. Louis, MO, United States). Stained cells were observed under an inverted microscope to evaluate virus replication in host cells according to the CPEs scale.

### 2.12 Antiviral assays (AVAs)

Antiviral assays (AVAs) of non-toxic concentrations of the blend were used to evaluate the antiviral activity against HCoV-OC43 in a cell-present environment. Tests were prepared with 24 h cell culture of HCT-8 cells at a density of 3 × 10^5^ cell/mL. The antiviral activity was assessed in three variants of the experiment: 1) Incubation of the cells with the blend 24 h before viral infection (OPTION 1), 2) Incubation of the cells with the blend simultaneously with the virus (OPTION 2), and 3) Incubation of the cells with the blend after viral infection (OPTION 3). HCT-8 cells were treated with the blend in several concentrations ranging from 50 to 150 μg/mL and then infected with 100 TCID_50/0.1_ mL of virus inoculum. The adsorption time for HCoV-OC43 was 90 min at 37 °C, 5 % CO_2_ atmosphere. After adsorption, the maintenance medium was discarded, and the cells were rinsed twice with PBS. Next, a fresh medium (OPTIONS 1 and 2) or blend solution (OPTION 3) was added to the cells. CPE were observed and evaluated under an inverted microscope using a five-point CPE scale as described above. Negative control cells were treated only with virus in maintenance medium. Positive controls included molnupiravir (50 μM) and interferon (0.01 μg/mL).

### 2.13 Statistical analysis

GraphPad Prism 9.5.0 software was used to analyze the data. The experimental results were expressed as mean ± standard deviation. One-way analysis of variance and a post-hoc test were used to analyze the statistical differences at the level of p < 0.05.

## 3 Results

### 3.1 Composition of the ELA blend

**Fig. 1:**
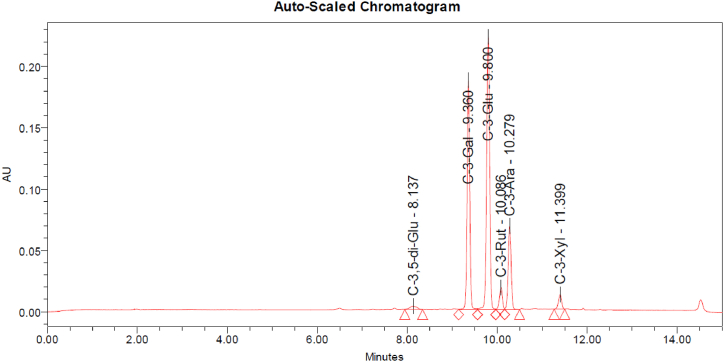
The chromatogram of the ELA blend with identified anthocyanin compounds. The blend showed the presence of compounds such as cyanidin-3,5-O-diglucoside (C-3,5-di-Glu), cyanidin 3-O-galactoside (C-3-Gal), cyanidin 3-O-glucoside (C-3-Glu), cyanidin 3-rutoside (C-3-Rut), cyanidin 3-O-arabinoside (C-3-Ara), and cyanidin 3-xyloside (C-3-Xyl).

The chromatographic analysis of the blend showed the presence of compounds such as cyanidin-3,5-O-diglucoside (C-3,5-di-Glu), cyanidin 3-O-galactoside (C-3-Gal), cyanidin 3-O-glucoside (C-3-Glu), cyanidin 3-rutoside (C-3-Rut), cyanidin 3-O-arabinoside (C-3-Ara), and cyanidin 3-xyloside (C-3-Xyl). The chromatogram is shown in Fig. 1. Based on the UV–Vis spectroscopy, we found 21.1 % (mass/mass; m/m) content of anthocyanins in the blend. The total phenolic content was quantified based on the Folin–Ciocalteu assay with caffeic acid as a calibrating curve. The phenolic content was 39.4 % (m/m).

### 3.2 ELA blend effect on SOD activity and cytokine production in A549 cells

**Fig. 2:**
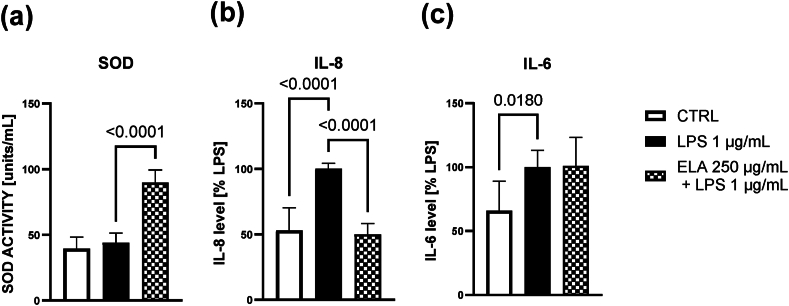
The effect of ELA blend (250 μg/mL) on (a) SOD activity, (b) IL-8 and (c) IL-6 production in LPS (1 μg/mL) stimulated A549 cells. After 4 h of preincubation with the ELA blend the cells were stimulated with LPS for another 24 h, and cell culture supernatants were collected for analysis. The results represent the mean ± SD from three independent experiments. Statistical analysis was performed with the ANOVA + post-hoc test. Comparisons with p < 0.05 were displayed.

In the first step, the non-toxic concentrations of the ELA blend were investigated. Cell viability was determined spectrophotometrically using the thiazolyl blue tetrazolium bromide (MTT) assay and lactate dehydrogenase (LDH) activity assay. The blend was non-toxic for A549 cells up to 500 μg/mL (Fig. 1S a-b in Supplementary data). Pretreatment of A549 cells with 250 μg/mL of the ELA blend resulted in a significant increase in SOD activity by 103.8 % (95%CI 76.04–131.5) compared to the LPS-treated group (Fig. 2 a), indicating enhanced cellular antioxidant defenses. Additionally, the ELA blend treatment led to a notable decrease (mean diff. – 50 %; 95%CI -66.1 to −34.0) in IL-8 production (Fig. 2 b), a pro-inflammatory cytokine involved in immune cell recruitment. However, no significant changes in IL-6 production were observed (Fig. 2 c).

### 3.3 ELA blend effect on SOD activity and cytokines production in EpiAirway™ tissues

**Fig. 3:**
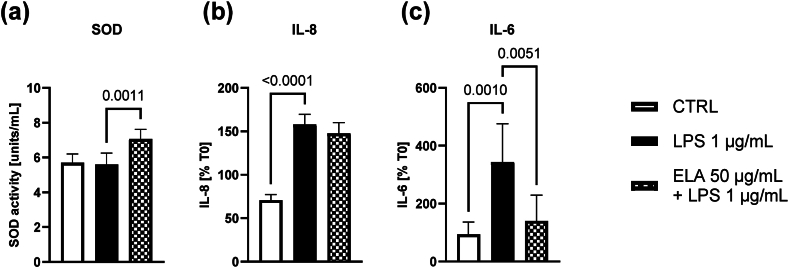
The effect of ELA blend (50 μg/mL) on (a) SOD activity, (b) IL-8 and (c) IL-6 cytokines production in LPS (1 μg/mL) stimulated EpiAirway™ tissues. After 4 h of preincubation with the ELA blend, the tissues were stimulated with LPS for 24 h, and culture media were collected for analysis. The results represent the mean ± SD. Statistical analysis was performed with the ANOVA + post-hoc test. Comparisons with p < 0.05 were displayed.

There were no changes in SOD activity in culture medium from LPS stimulated EpiAirway™ tissues. Statistically significant changes were observed for SOD activity (Fig. 3 a) at 24 h post-exposure only in the culture medium in group treated with ELA blend (mean diff. 26.2 % 95%CI 11.3 to 41.2). EpiAirway™ tissues reacted to stimulation with 1 μg/mL LPS with significant increases of IL-6 and IL-8 secretion in culture medium compared to unstimulated tissues (Fig. 3 b and c). There were no changes in the level of IL-8 in ELA blend + LPS-treated group compared to the level in the group treated with LPS alone (Fig. 3 b). Statistically significant decrease in IL-6 level in the culture medium was observed in ELA blend + LPS-treated group compared to the level of the LPS-treated group (mean diff. −41.3 % 95%CI -99.1 to −19.2) (Fig. 3 c).

### 3.4 ELA blend effect on gene expression in A549 cells and EpiAirway™ tissues

**Fig. 4:**
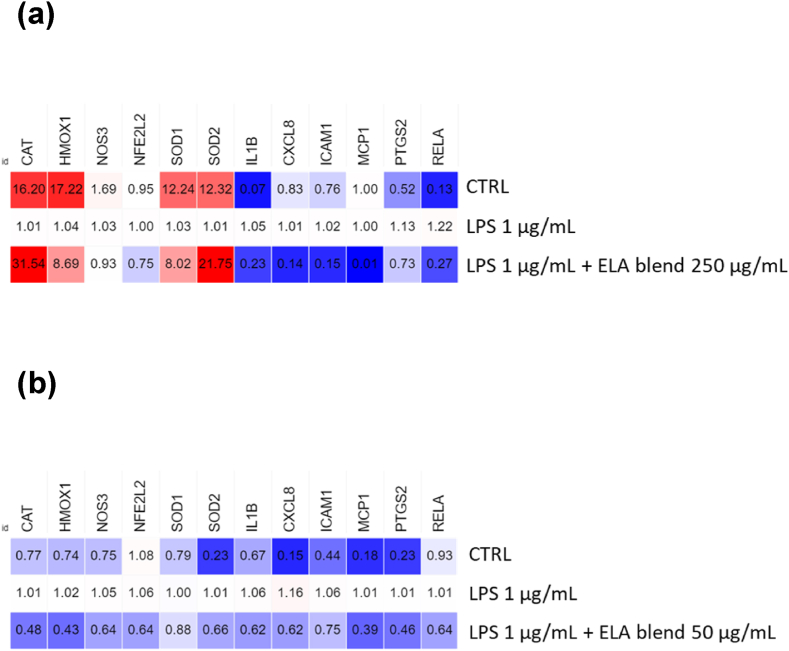
Real-time qRT-PCR verification of genes associated with oxidative stress and inflammation. The relative mRNA expression levels were determined against that of TBP, and the relative quantitation was calculated using 2^−ΔΔCt^ and compared between each group. Heatmaps, made in Morpheus (https://software.broadinstitute.org/morpheus), show the relative expression for: (a) A549 cells pretreated with ELA blend (250 μg/mL) for 2 h and stimulated with LPS (1 μg/mL) for the next 2 h, and (b) EpiAirway™ tissues pretreated with ELA blend (50 μg/mL) for 4 h and stimulated with LPS (1 μg/mL) for the next 24 h. The results represent the mean fold change (FC) from three independent experiments.

In A549 cells, the expression of several antioxidant genes was increased after ELA blend administration prior to LPS treatment. The expression of *CAT, HMOX1, SOD1*, and *SOD2* was observed to be significantly increased compared to the LPS groups, while the expression of *NOS3* and *NFE2L2* remained unchanged. Simultaneously, we investigated the expression levels of proinflammatory genes in A549 cells in response to ELA blend administration before LPS treatment. The expression of *IL1B, CXCL8, ICAM1, MCP1*, and *RELA* was significantly reduced in the cells treated with the ELA blend compared to the LPS group (Fig. 4 a). Following ELA blend treatment prior to LPS treatment in EpiAirway™ tissues, a significant downregulation of antioxidant genes expression was observed. Compared to the LPS-treated group, the expression levels of *CAT, HMOX1, NOS3, NFE2L2, SOD1*, and *SOD2* were significantly reduced. However, it is essential to take into account the possibility that the prolonged incubation time of 24 h had an effect on the dynamics of gene expression. It is possible that prolonged exposure to ELA blend treatment elicited distinct molecular mechanisms in the tissues, which resulted in the observed downregulation of antioxidant genes. This hypothesis is supported by the effects on expression levels observed in the negative control as well as the 24 h incubation of A549 cells with LPS and ELA blend (Supplementary data, Fig. S2), along with SOD activity tests (Fig. 3 a). It is conceivable that the most suitable interval of time for observing changes in gene expression triggered by ELA blend treatment in EpiAirway™ tissues may be different from the 24 h incubation period that was employed in this investigation.

Furthermore, the expression levels of proinflammatory genes were evaluated in EpiAirway™ tissues following ELA blend treatment prior to LPS treatment. Similar to A549 cells, the expression of *IL1B, CXCL8, MCP1, PTGS2*, and *RELA* was markedly reduced in ELA blend-treated tissues relative to LPS-stimulated tissues (Fig. 4 b).

### 3.5 ELA blend inhibits SARS-CoV2 S-protein RBD and ACE2 binding *in vitro*

**Fig. 5:**
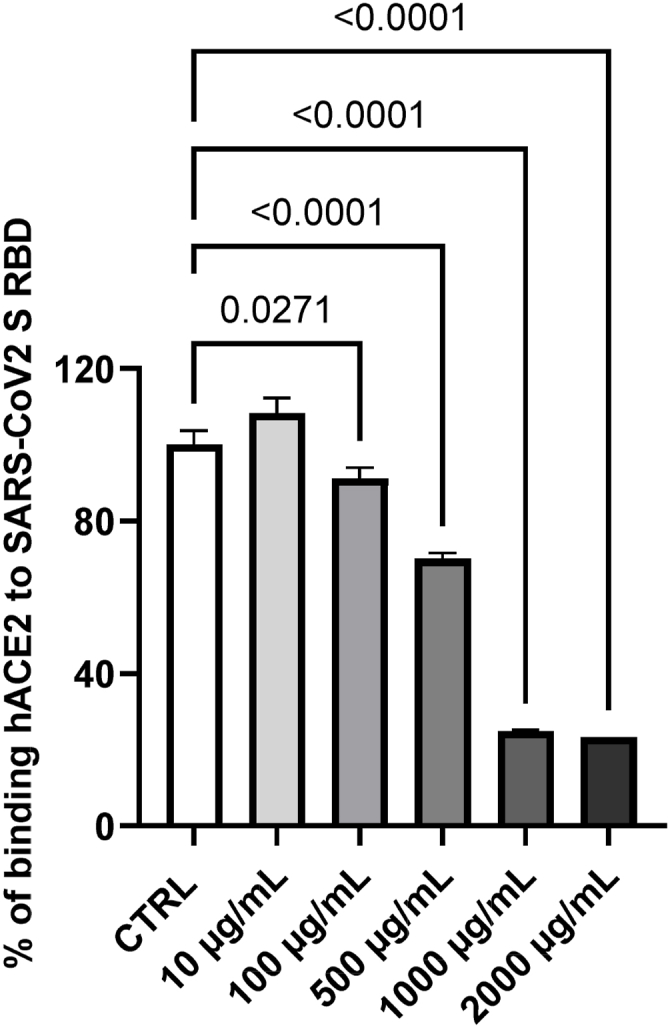
The effect of ELA blend on binding of recombinant human ACE2 (Angiotensin I Converting Enzyme 2) protein to SARS-CoV2 S protein receptor binding domain (RBD) in competitive ELISA. The blend was tested in five concentrations in duplicates, and the control contained only ACE2 without ELA blend. Statistically significant differences between the inhibitory effects of the applied blend concentrations and the control were assessed with ANOVA and a post hoc test. Comparisons with p < 0.05 were displayed.

We investigated the potential of the ELA blend in inhibiting the interaction between the spike protein RBD of SARS-CoV-2 and the ACE2 receptor in the presence of the ELA blend using competitive ELISA (Fig. 5). The blend effectively inhibited the interaction between spike protein RBD and the ACE2 receptor in a concentration-dependent manner.

### 3.6 *In vitro* anti-HCoV-OC43 activity of the ELA blend

To investigate the potential antiviral activity of the natural ELA blend, human coronavirus HCoV-OC43 was used. This virus is an adequate surrogate for SARS-CoV-2 because it also belongs to betacoronaviruses, infects the human respiratory system cells, and is transmitted in a similar way i.e. by droplets. In the first step, the non-toxic concentrations of the ELA blend for antiviral assays were investigated. HCT-8 cells at > 90 % confluence were treated with several concentrations of the blend ranging from 5 to 2000 μg/mL for 72 h at 37 °C and 5 % CO_2_. The fresh concentration of the blend (10 mg/mL) was prepared before each experiment in MilliQ water (heated to 50 °C) and filtered through a Millipore 0.22 μm filter. A series of dilutions were then made in an appropriate culture medium. Next, the cytotoxic effects (CTEs) were evaluated under an inverted microscope. The cytotoxic concentration (CC_50_), the concentration that causes death in 50 % of host cells, was estimated for the ELA blend. Results are presented in Fig. S3 (Supplementary data).

Antiviral activity of the ELA blend against the coronavirus HCoV-OC43 was examined *in vitro* in three experimental options (see Materials and methods) at the non-cytotoxic concentrations (Supplementary Fig. S3). After 4–5 days of incubation at 34 °C/5 % CO2 (the peak of virus production) viral CPEs were observed and assessed. Supplementary Fig. S4 presents how CPEs of HCoV-OC43 forms during several days.

**Fig. 6:**
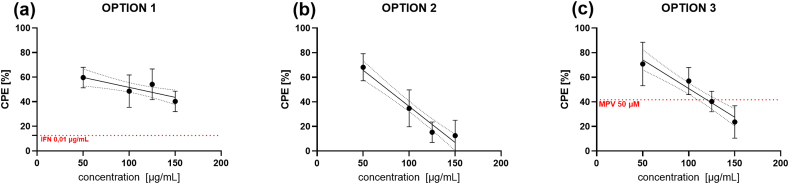
Antiviral activity of ELA blend against HCoV-OC43 in three experimental settings. a) OPTION 1, viral CPE in HCT-8 cells after 24 h preincubation with the blend; b) OPTION 2, viral CPE in HCT-8 cells after simultaneous incubation with the blend; c) OPTION 3, viral CPE in HCT-8 cells after incubation with the blend post viral infection. After 4–5 days of incubation (the peak of virus production) viral CPEs were observed and assessed. Results are shown as the mean percentage of the CPE from three independent experiments with triplicates each. The red line are thresholds of positive controls: molnupiravir (50 μM) and interferon (0.01 μg/mL), shown as a percentage of the cytopathic effect. (For interpretation of the references to color in this figure legend, the reader is referred to the Web version of this article.)

At the highest non-cytotoxic concentration of 150 μg/mL, the ELA blend decreased the viral titer by 59.7 % (95%CI - 66.1 to −53.3) in OPTION 1 when the blend was administered to the cells 24 h before virus infection; approximately 87.5 % (95%CI -97.1 to −77.9) in OPTION 2 when the blend was administered to the cells simultaneously with HCoV-OC43, and 76.4 % (95%CI -86.5 to −66.3) in OPTION 3 when the blend was added to the cells after virus infection. The results are presented in Fig. 6. The concentration inhibiting 50 % of virus replication (inhibitory concentration; IC_50_), together with the selectivity index (SI) calculated as a ratio of cytotoxic concentration and inhibitory concentration (CC_50_/IC_50_), are presented in Table 2. The best inhibitory effect was observed when the blend was administered simultaneously with the virus (IC_50_ = 76.7 μg/mL and SI = 5.8).

**Table 2:** Antiviral activity of ELA blend against HCoV-OC43.

	OPTION 1	OPTION 2	OPTION 3
CC_50_ (μg/ml)	447		
IC_50_ (μg/ml)	110.5	76.7	101.8
**SI**	**4.0**	**5.8**	**4.4**

SI = CC_50_/IC_50_.

## 4 Discussion

URTI are commonly caused by viruses, and most cases are self-limiting and do not require antibiotics [28]. Therapeutic interventions targeting viral infections remain a significant challenge [29]. Thus, herbal remedies have been used for prevention and treatment of viral respiratory illnesses [30]. The present study aimed to investigate the potential use of the standardized for polyphenols combination of LCK, AM, and EP as a therapeutic agent for URTI.

The effect observed in A549 cells and EpiAirway™ tissues demonstrated that the ELA blend significantly increased the activity of SOD, indicating improved cellular antioxidant defenses. Further analysis showed upregulation of antioxidant genes in A549 cells, including *CAT*, *HMOX1*, *SOD1*, and *SOD2*, after ELA blend treatment. The antioxidant effect of the ELA blend may be related to its high content of anthocyanins, such as C-3-Glu and C-3-Gal. It has been shown that C-3-Glu, the main component of LCK, enhances the expression of SOD2 in LPS-activated RAW 264.7 cells [31]. C-3-Gal, the most abundant anthocyanin in AM, reduces pulmonary fibrosis and significantly upregulates two important antioxidant mediators, NRF2 and HO1 [32]. This study's finding of an increase in the expression of antioxidant genes is consistent with other research that has examined how individual components of the ELA blend influence the expression of antioxidant genes. Li et al. [33] found that *Lonicera caerulea* L. polyphenols mitigate the intestinal environment imbalance caused by oxidative stress and liver injury caused by LPS in rats fed a high-fat diet by regulating the Nrf2/HO-1/NQO1 and MAPK pathways. Zapolska-Downar et al. [34] discovered that pretreatment of human aortic endothelial cells with AM fruit extract led to the activation of antioxidant genes including *HMOX1* and *NFE2L2*. Overall, rich in polyphenols, the ELA blend may be a promising antioxidant with potential health benefits.

Furthermore, the present study aimed to investigate the effects of the ELA blend on LPS-induced inflammation in A549 cells and EpiAirway™ tissues. The results demonstrated that the pretreatment with the ELA blend significantly reduced the production of IL-8 in A549 cells and IL-6 in EpiAirway™ tissues. The downregulation of proinflammatory genes such as *IL1B*, *CXCL8*, *ICAM1*, *MCP1*, and *RELA* in both A549 cells and EpiAirway™ tissues further supports the anti-inflammatory properties of the blend. These findings align with previous studies investigating the anti-inflammatory effects of individual components of the ELA blend, such as LCK and AM. For example, Appel et al. [35] reported that active components of AM inhibited the production of proinflammatory cytokines such as IL-6 and tumor necrosis factor-alpha (TNF-α) in LPS-stimulated macrophages. Additionally, Iwashima et al. [36] found that AM extract inhibited TNF-α-induced vascular endothelial inflammation through the downregulation of *IL1B*, *CXCL8*, *ICAM1* mRNA expression. Clinical studies also suggest that EP extract may serve as a natural anti-inflammatory agent for respiratory conditions such as bronchitis and sinusitis [37]. The observed anti-inflammatory effects indicate that the ELA blend could potentially be used as a therapeutic agent to alleviate inflammation in URTIs.

Since the severe acute respiratory syndrome coronavirus 2 (SARS-CoV-2) pandemic, interest in human coronaviruses and other respiratory viruses has been renewed. There has also been increased interest in potential drugs or supplements that could successfully treat or alleviate infections [38]. Polyphenols have been extensively studied for their potential benefits in reducing the severity and duration of upper respiratory tract infections. Quercetin, a polyphenol found in various fruits, has demonstrated efficacy in reducing the severity and duration of sickness in older patients with upper respiratory tract infections [39]. In addition, a double-standardized composition of AM and *Sambucus nigra* has shown effective inhibition of human influenza A virus (A/H1N1) and human betacoronavirus-1 (HCoV-OC43) replication [40]. Our findings showed a reduction in HCoV-OC43 titers in HCT-8 cells following 24 h of preincubation with the blend, indicating its potential to induce an antiviral state (e.g., by inducing interferon production) in the host cells (based on OPTION 1, selectivity indexes). Furthermore, simultaneous administration of the blend with HCoV-OC43 infection (OPTION 2) demonstrated a significant reduction in viral replication. Previous research has shown that EP increases IFN-α levels while simultaneously inhibiting IL-1β and TNF-α production [41]. IFN-α is crucial in the early antiviral response and is expressed as a first line of defense against viruses. The ability of viruses to counteract early antiviral responses, including those involving the host's IFN system, likely plays an important role in beta-coronaviruses virulence [42]. Upregulation of IFN could be a potential mechanism underlying the antiviral properties of the ELA blend. Notably, the ELA blendalso demonstrated antiviral activity when administered after HCoV-OC43 infection, suggesting its potential in mitigation ongoing infections. Our study focused on the early stages of the viral cycle, assessing the blend's activity after 24 h of incubation. However, further studies are warranted to determine the kinetics of the blend's action in the days following infection to fully understand its potential. Recent studies have highlighted the potential antiviral activity of anthocyanins against beta-coronaviruses, including SARS-CoV-2. C-3-Ara,a stable anthocyanin found in various fruits, including AM, has shown strong binding stability and interaction with key residues in the binding site of the coronavirus main protease (6lu7), suggesting its potential as an antiviral agent [43]. Elderberry, which contains C-3-Glu, has also been suggested to exert antiviral effects mediated by polyphenols interacting with viral glycoproteins [44]. While in-silico docking studies provide insights into the antiviral potential of anthocyanins, experimental validation is essential to confirm their therapeutic properties. These findings may provide insights into the mechanisms underlying the ELA blend's inhibitory effect on the binding of ACE2 to the spike RBD of SARS-CoV-2. However, further experimental validation is necessary to conclusively elucidate this phenomenon.

This study, while presenting promising results regarding the ELA blend's potential therapeutic effects on URTIs, including those caused by beta-coronaviruses, has several limitations that must be acknowledged. Our research primarily involved *in vitro* experiments using A549 cells and EpiAirway™ tissues, which, while valuable for initial screening, do not fully replicate the complexity of living organisms. The suggested mechanisms of action, including upregulation of antioxidant genes and downregulation of proinflammatory genes, were not directly measured in terms of viral entry. Thus, the antiviral effects inferred from binding assays need further substantiation through experiments that directly measure viral entry. While this methodology offers valuable insights into potential mechanisms of action, it does not definitively establish biological effects either *in vivo* or *in vitro*. Therefore, the conclusions drawn from previous docking studies regarding the impact of polyphenols from the ELA blend on viral entry remain speculative pending further validation through experimental data. Consequently, our conclusions regarding this process are theoretical rather than empirical. Moreover, the study's scope was restricted by the number of incubation protocols and *in vitro* models employed. Variations in incubation times and conditions may have influenced observed effects, underscoring the necessity for broader exploration of these parameters to comprehensively understand both the potential and limitations of ELA blend use.

In conclusion, our data showed that the polyphenol-standardized extracts combination of LCK, AM, EP in the ELA blend provides a range of health-promoting properties, including anti-inflammatory, antioxidant, and anti-beta-coronavirus effects. The anti-inflammatory and antioxidant properties of the individual components of the ELA blend have been reported in previous studies, and the present study provides evidence for the potential of the ELA blend as a therapeutic agent for URTI, including COVID-19. However, further studies are needed to investigate the efficacy of the ELA blend *in vivo* and to fully understand its effects on human health. If proven effective, the ELA blend may offer a natural and safe alternative to conventional therapies for respiratory diseases.

## Funding

This study was supported by the European Union through the European Regional Development Fund under the Smart Growth Operational Programme. Project POIR.01.01.01-00-1206/20 is implemented under The National Center for Research and Development (Narodowe Centrum Badań i Rozwoju) call for proposals: “Fast Track”.

## Availability of data and materials

Data will be made available on request

During the preparation of this work the authors used QuillBot tool in order to enhance the quality of the text by ensuring clarity and coherence. After using this tool/service, the authors reviewed and edited the content as needed and take full responsibility for the content of the publication.

## CRediT authorship contribution statement

**Katarzyna Zima:** Writing – review & editing, Writing – original draft, Supervision, Methodology, Investigation, Formal analysis, Conceptualization. **Barbara Khaidakov:** Writing – review & editing, Supervision, Formal analysis. **Marta Sochocka:** Writing – review & editing, Writing – original draft, Methodology, Investigation. **Michał Ochnik:** Writing – review & editing, Methodology, Investigation. **Krzysztof Lemke:** Writing – review & editing, Funding acquisition, Conceptualization. **Paulina Kowalczyk:** Writing – review & editing, Writing – original draft, Visualization, Supervision, Methodology, Investigation, Formal analysis, Conceptualization.

## Declaration of competing interest

The authors declare the following financial interests/personal relationships which may be considered as potential competing interests:Krzysztof Lemke reports financial support was provided by National Center for Research and Development. Paulina Kowalczyk has patent #P.444627; P.446422 pending to Licensee. Katarzyna Zima has patent #P.444627; P.446422 pending to Licensee. Krzysztof Lemke has patent #P.444627; P.446422 pending to Licensee. If there are other authors, they declare that they have no known competing financial interests or personal relationships that could have appeared to influence the work reported in this paper.

## References

[1] (2020). Urgent Health Challenges for the Next Decade.

[2] Jain N., Lodha R., Kabra S.K. (2001). Upper respiratory tract infections. Indian J. Pediatr..

[3] Shahan B., Barstow C., Mahowald M. (2019). Respiratory conditions: upper respiratory tract infections. FP Essent.

[4] Orsavová J., Sytařová I., Mlček J., Mišurcová L. (2022). Phenolic compounds, vitamins C and E and antioxidant activity of edible honeysuckle berries (Lonicera caerulea L. Var. kamtschatica pojark) in relation to their origin. Antioxidants.

[5] Kang D., Kim D. (2021). Antioxidant effect of Lonicera Caerulea on heat stress-treated male mice. Journal of Animal Reproduction and Biotechnology.

[6] An M., Eo H., Son H., Geum N., Park G., Jeong J. (2020). Anti inflammatory effects of leaf and branch extracts of honeyberry (Lonicera caerulea) on lipopolysaccharide stimulated RAW264.7 cells through ATF3 and Nrf2/HO 1 activation. Mol. Med. Rep..

[7] Lu H., Zhang L., Huang H. (2016). Study on the isolation of active constituents in Lonicera japonica and the mechanism of their anti-upper respiratory tract infection action in children. Afr. Health Sci..

[8] Minami M., Nakamura M., Makino T. (2019). Effect of Lonicera caerulea var. emphyllocalyx extracts on murine Streptococcus pyogenes infection by modulating immune system. BioMed Res. Int..

[9] Ren Y., Frank T., Meyer G., Lei J., Grebenc J.R., Slaughter R. (2022). Potential benefits of black chokeberry (Aronia melanocarpa) fruits and their constituents in improving human health. Molecules.

[10] Eggers M., Jungke P., Wolkinger V., Bauer R., Kessler U., Frank B. (2022). Antiviral activity of plant juices and green tea against <scp>SARS‐CoV</scp> ‐2 and influenza virus. Phytother Res..

[11] Benson J.M., Pokorny A.J., Rhule A., Wenner C.A., Kandhi V., Cech N.B. (2010). Echinacea purpurea extracts modulate murine dendritic cell fate and function. Food Chem. Toxicol..

[12] Enany M., Algammal A.E.A., Solimane R., El Sissi A., Hebashy A. (2017). Evaluation of Echinacea immunomodulatory effect on the immune response of broiler chickens. Suez Canal Veterinary Medicine Journal SCVMJ.

[13] Kim H.-R., Oh S.-K., Lim W., Lee H.K., Moon B.-I., Seoh J.-Y. (2014). Immune enhancing effects of Echinacea purpurea root extract by reducing regulatory T cell number and function. Nat. Prod. Commun..

[14] Goel V., Chang C., Slama J.V., Barton R., Bauer R., Gahler R. (2002). Alkylamides of Echinacea purpurea stimulate alveolar macrophage function in normal rats. Int. Immunopharm..

[15] Goel V., Chang C., Slama J.V., Barton R., Bauer R., Gahler R. (2002). Echinacea stimulates macrophage function in the lung and spleen of normal rats. J. Nutr. Biochem..

[16] Sharma M., Arnason J.T., Burt A., Hudson J.B. (2006). Echinacea extracts modulate the pattern of chemokine and cytokine secretion in rhinovirus‐infected and uninfected epithelial cells. Phytother Res..

[17] Schumacher A., Friedberg K.D. (1991). [The effect of Echinacea angustifolia on non-specific cellular immunity in the mouse]. Arzneimittelforschung.

[18] Gan X.-H., Zhang L., Heber D., Bonavida B. (2003). Mechanism of activation of human peripheral blood NK cells at the single cell level by Echinacea water soluble extracts: recruitment of lymphocyte–target conjugates and killer cells and activation of programming for lysis. Int. Immunopharm..

[19] Kolev E., Mircheva L., Edwards M.R., Johnston S.L., Kalinov K., Stange R. (2022). Echinacea purpurea for the long-term prevention of viral respiratory tract infections during covid-19 pandemic: a randomized, open, controlled, exploratory clinical study. Front. Pharmacol..

[20] Cohen H.A., Varsano I., Kahan E., Sarrell E.M., Uziel Y. (2004). Effectiveness of an herbal preparation containing Echinacea, propolis, and vitamin C in preventing respiratory tract infections in children. Arch. Pediatr. Adolesc. Med..

[21] David S., Cunningham R. (2019). Echinacea for the prevention and treatment of upper respiratory tract infections: a systematic review and meta-analysis. Compl. Ther. Med..

[22] Zielińska A., Siudem P., Paradowska K., Gralec M., Kaźmierski S., Wawer I. (2020). Aronia melanocarpa fruits as a rich dietary source of chlorogenic acids and anthocyanins: 1H-nmr, HPLC-DAD, and chemometric studies. Molecules.

[23] Banach M., Wiloch M., Zawada K., Cyplik W., Kujawski W. (2020). Evaluation of antioxidant and anti-inflammatory activity of anthocyanin-rich water-soluble Aronia dry extracts. Molecules.

[24] Ochmian I., Grajkowski J., Skupien K. (2010). Yield and chemical composition of blue honeysuckle fruit depending on ripening time. Bulletin UASVM Horticulture.

[25] Livak K.J., Schmittgen T.D. (2001). Analysis of relative gene expression data using real-time quantitative PCR and the 2−ΔΔCT method. Methods.

[26] Ramakrishnan M.A. (2016). Determination of 50% endpoint titer using a simple formula. World J. Virol..

[27] Lambert F., Jacomy H., Marceau G., Talbot P J. (2008). Titration of human coronaviruses using an immunoperoxidase assay. JoVE.

[28] Griffiths M. (2017). Ruling out antibiotics. Nurs. Stand..

[29] Khatun S., Putta C.L., Hak A., Rengan A.K. (2023). Immunomodulatory nanosystems: an emerging strategy to combat viral infections. Biomaterials and Biosystems.

[30] Mammari N., Albert Q., Devocelle M., Kenda M., Kočevar Glavač N., Sollner Dolenc M. (2023). Natural products for the prevention and treatment of common cold and viral respiratory infections. Pharmaceuticals.

[31] Wu S., Yano S., Chen J., Hisanaga A., Sakao K., He X. (2017). Polyphenols from Lonicera caerulea L. Berry inhibit LPS-induced inflammation through dual modulation of inflammatory and antioxidant mediators. J. Agric. Food Chem..

[32] Cui Y., Zhao J., Chen J., Kong Y., Wang M., Ma Y. (2022). Cyanidin-3-galactoside from Aronia melanocarpa ameliorates silica-induced pulmonary fibrosis by modulating the TGF-β/mTOR and NRF2/HO-1 pathways. Food Sci. Nutr..

[33] Li B., Cheng Z., Sun X., Si X., Gong E., Wang Y. (2020). Lonicera caerulea L. Polyphenols alleviate oxidative stress‐induced intestinal environment imbalance and lipopolysaccharide‐induced liver injury in HFD‐fed rats by regulating the Nrf2/HO‐1/NQO1 and MAPK pathways. Mol. Nutr. Food Res..

[34] Zapolska-Downar D., Bryk D., Małecki M., Hajdukiewicz K., Sitkiewicz D. (2012). Aronia melanocarpa fruit extract exhibits anti-inflammatory activity in human aortic endothelial cells. Eur. J. Nutr..

[35] Appel K., Meiser P., Millán E., Collado J.A., Rose T., Gras C.C. (2015). Chokeberry (Aronia melanocarpa (Michx.) Elliot) concentrate inhibits NF-κB and synergizes with selenium to inhibit the release of pro-inflammatory mediators in macrophages. Fitoterapia.

[36] Iwashima T., Kudome Y., Kishimoto Y., Saita E., Tanaka M., Taguchi C. (2019). Aronia berry extract inhibits TNF-α-induced vascular endothelial inflammation through the regulation of STAT3. Food Nutr. Res..

[37] Schapowal A., Klein P., Johnston S.L. (2015). Echinacea reduces the risk of recurrent respiratory tract infections and complications: a meta-analysis of randomized controlled trials. Adv. Ther..

[38] Low Z., Lani R., Tiong V., Poh C., AbuBakar S., Hassandarvish P. (2023). COVID-19 therapeutic potential of natural products. Int. J. Mol. Sci..

[39] Heinz S.A., Henson D.A., Austin M.D., Jin F., Nieman D.C. (2010). Quercetin supplementation and upper respiratory tract infection: a randomized community clinical trial. Pharmacol. Res..

[40] Ochnik M., Franz D., Sobczyński M., Naporowski P., Banach M., Orzechowska B. (2022). Inhibition of human respiratory influenza A virus and human betacoronavirus-1 by the blend of double-standardized extracts of Aronia melanocarpa (michx.) elliot and Sambucus nigra L. Pharmaceuticals.

[41] Zhai Z., Liu Y., Wu L., Senchina D.S., Wurtele E.S., Murphy P.A. (2007). Enhancement of innate and adaptive immune functions by multiple Echinacea species. J. Med. Food.

[42] Bastard P., Zhang Q., Zhang S.-Y., Jouanguy E., Casanova J.-L. (2022). Type I interferons and SARS-CoV-2: from cells to organisms. Curr. Opin. Immunol..

[43] Messaoudi O., Gouzi H., El-Hoshoudy A.N., Benaceur F., Patel C., Goswami D. (2021). Berries anthocyanins as potential SARS-CoV–2 inhibitors targeting the viral attachment and replication; molecular docking simulation. Egyptian Journal of Petroleum.

[44] Torabian G., Valtchev P., Adil Q., Dehghani F. (2019). Anti-influenza activity of elderberry (Sambucus nigra). J. Funct.Foods.

