# Supplementary material for: Exploring the potency of polyphenol-rich blend from Lonicera caerulea var. Kamtschatica sevast., Aronia melanocarpa, and Echinacea purpurea: Promising anti-inflammatory, antioxidant, and antiviral properties
Source: Heliyon. 2024 Aug 2;10(15):e35630. doi: 10.1016/j.heliyon.2024.e35630 (PMC11336823; doi:10.1016/j.heliyon.2024.e35630)
Supplement: Multimedia component 1 [file mmc1.docx]

**Supplementary materials**

***Supplementary Fig. S1.*** *Cytotoxicity of ELA blend against A549 cells and EpiAirway^TM^. A)* *A549 cells (8x10^4^/mL) were incubated for 24h at 37 °C, without any addition of the blend (as a control) and with varied concentrations of the blend (3.9-1000 μg/mL). Next, 20 µL of filter sterilized MTT (5 mg/mL) in phosphate buffered saline (PBS) was added to each well and incubated at 37 °C for 4h. The medium with MTT was removed, and the formed formazan crystals were dissolved in 100 µL DMSO (VWR International, Radnor, PA, USA). The absorbance was measured at 570 nm using EnVision 2103 Multilabel Reader (PerkinElmer, Waltham, MA, USA).* *Results were expressed as a % of untreated control. B) A549 Cells (8x10^4^/mL) were incubated for 24h at 37 °C, without any addition of the blend (as a control) and with varied concentrations of the blend (3.9-1000 μg/mL). Next, the lactate dehydrogenase (LDH) cell assay (CytoTox 96^®^ Non-Radioactive Cytotoxicity, Promega, Madison, WI, USA) was performed according to the manufacturer protocol. The absorbance was measured at 520 nm using EnVision 2103 Multilabel Reader (PerkinElmer, Waltham, MA, USA).* *Results were expressed as an optical density (OD).* *C) EpiAirway^TM^ tissues were incubated for 24h at 37 °C, without any addition of the blend (as a control) and with four concentrations of the blend (5-50 μg/mL). Next, the lactate dehydrogenase (LDH) cell assay (CytoTox 96^®^ Non-Radioactive Cytotoxicity, Promega, Madison, WI, USA) was performed in apical washings according to the manufacturer protocol. The absorbance was measured at 520 nm using EnVision 2103 Multilabel Reader (PerkinElmer, Waltham, MA, USA). Results were expressed as an optical density (OD).*


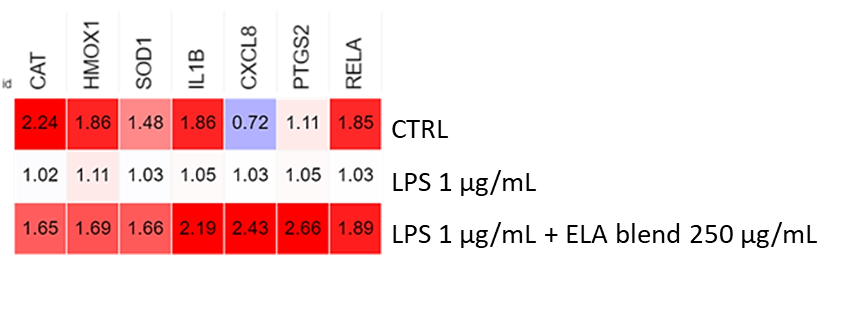


***Supplementary Fig. S2.*** *Real-time qRT-PCR verification of genes associated with oxidative stress and inflammation. The relative mRNA expression levels were determined against that of TBP, and the relative quantitation was calculated using 2^−ΔΔCt^ and compared between each group. Heatmap, made in Morpheus (https://software.broadinstitute.org/morpheus), shows the relative expression for A549 cells pretreated with ELA blend (250 µg/mL) for 2h and stimulated with LPS (1 µg/mL)* *for the next 24h. The results represent the mean fold change (FC) from three independent experiments.*

***Supplementary Fig. S3.*** *Cytotoxicity of ELA blend against HCT-8 cells. HCT-8 cells were treated with several concentrations of the blend (in a range of 5-2000 μg/mL) for 72h, at 37֯C, 5% CO_2_. Next, the cytotoxic effects (CTEs) were evaluated under an inverted microscope. The negative control was untreated cells cultured only with medium. The viability was measured by evaluating morphological changes observed by an inverted microscope assessed by a four‐point cytotoxic effects (CTEs) scale, where 0—lack of visible CTEs in the cells; 1—CTEs in up to 25% of the cells; 2—CTEs in up to 50% of the cells; 3—CTEs in up to 75% of the cells; and 4—CTEs in up to 100% of the cells. The negative control was untreated cells cultured only with maintenance medium. The experiment was performed three times in at least three independent repetitions. Estimated cytotoxic concentration (CC50), a concentration that causes death to 50% of host cells, was CC50 = 447μg/mL. The blend showed a complete lack of toxicity for concentrations ≤ 150 μg/mL (CTE=0).*


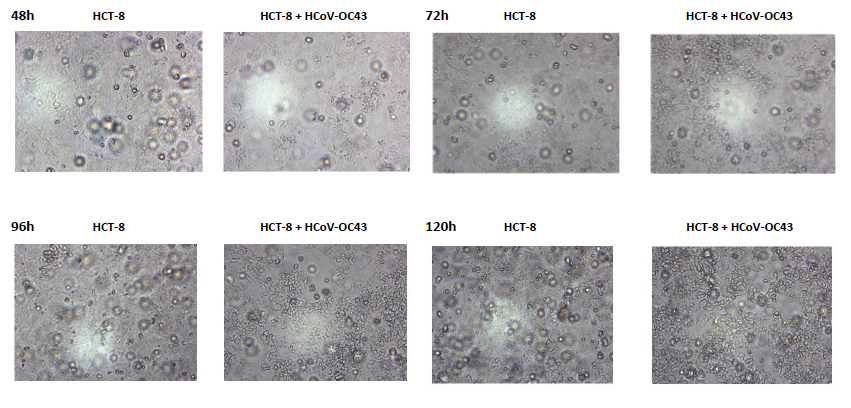


***Supplementary Fig. S4.*** *CPEs of HCoV-OC43 formed during 5 days of incubation. Inverted microscope (x100). HCT-8 cells at density 3 x 105 cell/mL were infected with 100 TCID50/0.1 mL of HCoV-OC43. The cells were then incubated for up to 5 days at 34 °C/ 5% CO2 in RPMI 1640 containing 2% FBS. CPEs were monitored by light inverted microscope at the indicated days post-infection.*
